# Measuring the mechanical properties of plant cells by combining micro-indentation with osmotic treatments

**DOI:** 10.1093/jxb/erv135

**Published:** 2015-04-07

**Authors:** Alain Weber, Siobhan Braybrook, Michal Huflejt, Gabriella Mosca, Anne-Lise Routier-Kierzkowska, Richard S. Smith

**Affiliations:** ^1^Institute of Plant Sciences, University of Bern, Altenbergrain 21, CH-3013 Bern, Switzerland; ^2^Sainsbury Laboratory, Bateman Street, Cambridge, CB2 1LR, UK; ^3^Max Planck Institute for Plant Breeding Research, Carl-von-Linné-Weg 1050829 Köln, Cologne, Germany

**Keywords:** BY-2, cell wall elasticity, cellular force microscopy, finite-element method, mechanical modelling, micro-indentation, osmotic treatments, sensitivity analysis, turgor pressure.

## Abstract

Growth in plants results from the interaction between genetic and signalling networks and the mechanical properties of cells and tissues. There has been a recent resurgence in research directed at understanding the mechanical aspects of growth, and their feedback on genetic regulation. This has been driven in part by the development of new micro-indentation techniques to measure the mechanical properties of plant cells *in vivo*. However, the interpretation of indentation experiments remains a challenge, since the force measures results from a combination of turgor pressure, cell wall stiffness, and cell and indenter geometry. In order to interpret the measurements, an accurate mechanical model of the experiment is required. Here, we used a plant cell system with a simple geometry, *Nicotiana tabacum* Bright Yellow-2 (BY-2) cells, to examine the sensitivity of micro-indentation to a variety of mechanical and experimental parameters. Using a finite-element mechanical model, we found that, for indentations of a few microns on turgid cells, the measurements were mostly sensitive to turgor pressure and the radius of the cell, and not to the exact indenter shape or elastic properties of the cell wall. By complementing indentation experiments with osmotic experiments to measure the elastic strain in turgid cells, we could fit the model to both turgor pressure and cell wall elasticity. This allowed us to interpret apparent stiffness values in terms of meaningful physical parameters that are relevant for morphogenesis.

## Highlight

A combination of osmotic treatments, micro-indentation with cellular force microscopy, and inverse finite-element modelling gives an estimate for both turgor pressure and cell wall elasticity in plant cells.

## Introduction

Growth and morphogenesis result from the controlled expansion of individual cells coordinated at the tissue and organ level. In plant tissue, cells cannot move with respect to each other, and the development of complex shapes must rely instead almost entirely on precise mechanisms to regulate cell expansion and anisotropy. Although much research has been focused on elucidating the genetic and biochemical signalling aspects of morphogenesis, there is a growing interest in understanding the mechanical aspects of growth, and their feedback on genetic regulation (Hamant *et al.*, 2008; Heisler *et al.*, 2010; Peaucelle *et al.*, 2011). Growth itself is a mechanical process, and the output of molecular signalling networks must ultimately be translated into the physical properties of plant cells and tissues. Potential targets are turgor pressure and mechanical properties of the cell wall, because they determine growth rate and growth direction (Cosgrove, 1993, 2005; Goriely *et al.*, 2008; Geitmann and Ortega, 2009). In order to study the signalling pathways that regulate growth and morphogenesis, it is therefore necessary to measure these quantities at a cellular level.

Turgor pressure and the bulk elastic modulus of a plant cell can be measured with a pressure probe (Tomos and Leigh, 1999). This device is made from a microcapillary that is attached to a pressure transducer. While the probe is inserted into a cell, turgor is found as the pressure that prevents cell sap from entering the capillary. The pressure probe measures turgor in a direct way but causes irreversible damage to the cell and is limited in terms of the number and size of the cells that can be measured. A more recent, less-invasive approach for measuring elastic and viscoelastic properties of plant cells are indentation methods. These include atomic force microscopy (AFM) (Milani *et al.*, 2011; Peaucelle *et al.*, 2011; Sampathkumar *et al.*, 2014), cellular force microscopy (CFM) (Felekis *et al.*, 2011; Routier-Kierzkowska *et al.*, 2012), microcompression (Wang *et al.*, 2004, 2006), and other single-point indentation systems (Lintilhac *et al.*, 2000; Hayot *et al.*, 2012). Indentation methods are based on the idea of measuring the force that is needed to displace the surface of a sample by a given distance. The resulting force versus indentation curves can then be used to calculate the apparent stiffness of a sample. For a review of indentation methods used on plants, see Geitmann (2006), Routier-Kierzkowska and Smith (2013), and Milani *et al.* (2013).

Interpreting the results is a major issue with indentation studies because the methods do not measure a specific physical property. Depending on probe size, indentation depth, and indentation speed, the measurement can reflect a combination of turgor pressure, cell wall elasticity and viscoelasticity, cell geometry, indenter geometry, and boundary conditions. In order to untangle the effect of specific physical properties, it is necessary to solve an inverse mechanical problem, i.e. to find model parameters that best fit the data.

Several models have been proposed that describe indentation experiments at different scales. A mathematical model that is often used to interpret data from AFM experiments is the Hertz model (Lin *et al.*, 2007). This assumes that indentations are small enough to only probe elastic properties of the cell wall. In particular, the stiffness does not depend on turgor pressure, which has been demonstrated in plants for small (substantially less than a cell wall thickness) indentations on the shoot apical meristem (Milani *et al.*, 2011). The Hertz model is appealing because the relationship between force, indentation, and material properties can be expressed by a formula. It is, however, limited to homogeneous and isotropic materials and a small number of contact geometries. In turgid plant cells, the homogeneity requirement (in depth) is violated unless the indentation depth is very small (Milani *et al.*, 2011), and even for very small indentations, in-plane spatial differences in stiffness have been observed (Sampathkumar *et al.*, 2014). As the depth of the indentation increases, turgor pressure starts to play a role up to the point where it dominates the apparent stiffness. In onion epidermis, the apparent stiffness has been shown to vary up to 6-fold depending on the turgidity of the tissue (Routier-Kierzkowska *et al.*, 2012). In this case, the tip size (2–3 μm in diameter) and indentation depth (1–2 μm) were in the same order of magnitude as the cell wall thickness (~1 μm). Recently, a model for the indentation of an isotropic spherical and ellipsoidal shell with a point probe was proposed (Vella *et al.*, 2012*a*, *b*). Unlike the Hertz model, this includes turgor pressure and can therefore be used to interpret large indentations on turgid cells with isotropic cell walls. In the asymptotic case of very large indentations on a highly pressurized shell, the reaction force was found to depend only on pressure, indentation depth, and radius of the shell; however, their formula for smaller indentations also depends on cell wall elasticity. The dominating influence of turgor pressure was also found in a study by Lintilhac *et al.* (2000) when using a glass bead of 50–500 μm diameter to indent onion epidermal cells. By using an optical system to observe the contact patch, it was found that contact force is the product of turgor pressure and the projected contact area, demonstrating a significant role for indenter geometry in this system. This relationship was later reproduced on suspension-cultured tomato cells (Wang *et al.*, 2006) when performing large indentations with a flat probe (microcompression).

These studies show that different indentation techniques depend to varying degrees on different experimental, mechanical, and geometrical parameters. Here, we examined which parameters affect micro-indentation measurements when indentations are performed on thin-walled cells with a small probe (1 μm diameter) and indentation depth is about 15% of the cell radius (~2.5 µm). We were particularly interested in determining to what extent micro-indentation is sensitive to cell wall elasticity and turgor pressure. To answer this question, we performed CFM experiments on a single plant cell system with very simple geometry, tobacco Bright Yellow-2 (BY-2) cells (Nagata *et al.*, 1992). To interpret the results of our measurements, we developed a mechanical finite-element simulation of the experiment and studied the influence of the various parameters on the predicted reaction force (Deng *et al.*, 2011; Forouzesh *et al.*, 2013; Vogler *et al.*, 2013). Based on this parameter sensitivity analysis, we identified a minimal set of relevant parameters and showed that micro-indentation experiments alone were not sufficient to fully constrain the model and extract both cell wall elasticity and turgor pressure. We therefore complemented CFM indentations with measurements of cell deformation under osmotic treatments, which are influenced more directly by cell wall elasticity. Here, we present the results of our sensitivity analysis and a new method designed to reliably measure turgor pressure and cell wall elasticity in plant cells, which combines CFM, osmotic manipulations, and inverse mechanical modelling.

## Materials and methods

### Plant material

Tobacco (*Nicotiana tabacum*) BY-2 cells (DSMZ, Germany) were grown at 25 °C in the dark, with constant shaking at 50rpm. Cells were subcultured each week as 10% dilutions into fresh medium. Culture medium composition was as follows: 1× MS salts (Duchefa Biochemie, The Netherlands), 3% sucrose, 100mg l^–1^ of myo-inositol, 1mg l^–1^ of thiamin, and 0.2mg l^–1^ of 2,4-dichlorophenoxyacetic acid, at pH 5.8.

### Osmotic shrinkage assay

BY-2 cells (2–4 d post-subculture) were pipetted onto tissue culture frost-treated plastic dishes and rinsed with either water or 0.2M mannitol solution to wash off non-adhering cells. The cells were then immediately covered with either 0.2M mannitol solution or water and given 15min to equilibrate the turgor pressure. An inverted microscope (Olympus IMT-2) with a ×20 objective was used to focus on a group of cells and to take a focus stack of pictures. Finally, the medium was replaced by 0.55M mannitol solution to trigger plasmolysis. Once the membrane visibly retracted from the cell wall, another focus stack was taken from the same group of cells. The length of each cell in both stacks was measured with ImageJ and the PointPicker plug-in. The longitudinal strain *ε*_*l*_ was calculated from the pressurized length *l* of a cell in 0 or 0.2M mannitol solution and from the plasmolysed length *L* of the same cell as *ε*_*l*_=ln(*l*/*L*).

### Incipient plasmolysis determination

BY-2 cells (1–2 d post-subculture) were concentrated by passage of the culture through 40 μm sieves. Approximately 100 μl volumes of concentrated cells were placed in the following mannitol solutions and imaged with confocal microscopy after 15min of incubation: first replicate: water (0), 0.2, 0.3, 0.4, 0.45, 0.5, and 0.6M; second replicate with finer steps: 0.25, 0.275, 0.3, 0.325, 0.35, 0.375, 0.4, 0.425, and 0.45M. After the incubation, a 100 μl aliquot of the cells was added to 10 μl of 1× Calcofluor white (Sigma) and 0.1 μl of 1M NaOH on a slide and covered with a cover slip. Cells were imaged using a Leica DMR XE7 with a ×60 objective. Images were collected using a 405 UV laser for excitation of Calcofluor white-stained cell walls (emission collection at 500–520nm), and differential interference contrast microscopy for visualization of plasmolysis. Incipient plasmolysis was determined as the point at which the membrane separated from the cell wall in 50% of the cells. In the first replicate, this was observed at an osmolarity of 0.4M. In the second finer replicate, this was further quantified to be between 0.4M and 0.425M.

### Cell preparation for CFM

For the CFM experiment, we pipetted cells (2–6 d post-subculture) onto a glass slide that had previously been coated with a bio-adhesive (BIOBOND; BBInternational). The slide was then immediately rinsed with solution to wash off non-adhering cells. Finally, the slide was put into a glass dish and submerged in either pure water or 0.2M mannitol solution. After 30min, the samples were examined under a cellular force microscope.

## Results

### Cellular Force Microscopy

**Fig. 1.**
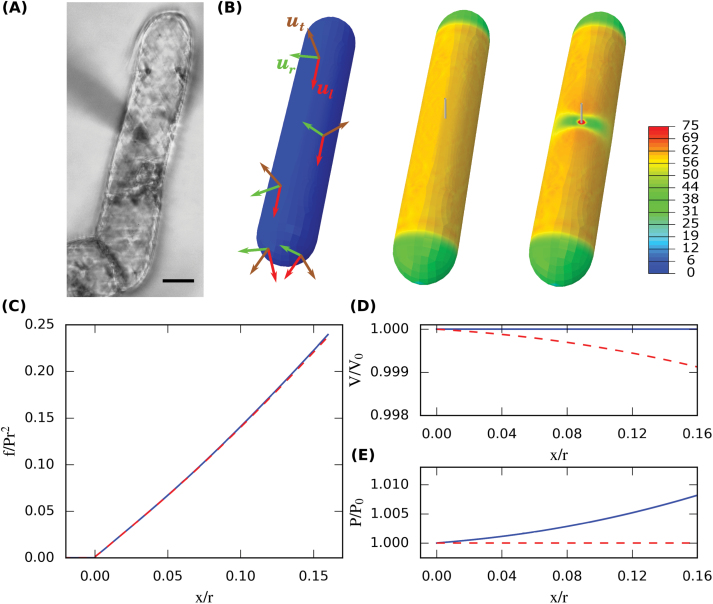
Mechanical modelling of CFM on a tobacco BY-2 cell. (A) Micro-indentation on a tobacco BY-2 cell seen from below on an inverted microscope. Note the shadow of the probe attached to the force sensor as it approaches the sample from above. Bar, 20 μm. (B) Finite-element simulation of the indentation experiment using the parameters *l*/*r*=150/15, *t*/*r*=0.25/15, *x*/*r*=2.4/15, *s*/*r*=0.5/15, *ε*_*r*_=0.025, *ε*_*l*_=0.05, *β*=2, and *υ*_*rl*=_0.4 (see Eq. 5 in text). The undeformed cell (left) is first pressurized (middle) and then indented (right). Material properties are homogeneous but depend on three principal material directions (left). The heatmap reports maximum principal stress (in MPa) within the shell. (C–E) Simulated force versus indentation curves assuming either constant pressure (red dashed line) or constant volume (blue solid line) during indentation (C). Depending on this assumption, either the volume decreases (D) or the pressure increases (E) during indentation.

CFM is a combination of three (*x*-*y*-*z*) robotic positioners (SLC-2475; SmarAct GmbH) and a MEMS-based force sensor (FT S-540; FemtoTools) to which a tungsten probe (T-4–22; Picoprobe) is attached. The positioners have a resolution of 1nm, and the force sensors have a sensitivity of 0.3 μN at 1000 Hz. The robot itself is mounted on an inverted microscope (Olympus IMT-2). More details about the set-up can be found in Routier-Kierzkowska *et al.*, (2012). Indentation experiments were performed as follows. First, the probe was manually positioned above the middle of a cell in order to ensure perpendicular contact between the cell and the probe. If the probe was positioned away from the middle of the cell, the cell would slip away during indentation. Indentation was always performed at least two radii distance from the end of a cell to minimize any effect from the hemispherical end geometry (Fig. 1A and Supplementary Fig. S2 at *JXB* online). Next, we ran an indentation program based on a closed-loop control of the robot positioner. The procedure consisted of three iterations to assess repeatability. Each iteration was a combination of a coarse approach followed by a fine approach. During the coarse approach, the probe moved towards the sample with a step size of 100nm to detect the surface. The contact between probe and surface was found based on the increment of force between each step, i.e. when a stiffness threshold was reached. Once contact was detected for the first time, the probe retracted by a given distance (~3 μm). This guaranteed that the fine approach would contain force versus *z*-position data from before and after contact. In particular, it allowed us to re-estimate the point of contact during post-processing and to distinguish between the stiffness of the sample and the stiffness of the water meniscus (Routier-Kierzkowska *et al.*, 2012).

The fine approach then made the actual measurements, and involved moving the probe down by a predefined distance at a step size of 60nm until the maximum desired indentation depth was reached. Data was also recorded as the probe was retraced at the same step size. Further details on the calibration (Felekis *et al.*, 2012) and post-processing of data can be found in the Supplementary Information at *JXB* online.

### Mechanical model of a BY-2 cell

In order to interpret the results of force measurements on BY-2 cells, we developed a mechanical model of the micro-indentation experiment. The model was defined in terms of continuum mechanics and described the indentation of a single turgid cell. The geometry of the non-turgid cell was idealized as a cylindrical shell capped by two hemispherical shells (Fig. 1B), a realistic approximation of the tobacco BY-2 cells used in our experiments. The shell was assigned a uniform thickness and homogeneous material properties. We used a linear orthotropic (i.e. anisotropic, with different properties along three mutually orthogonal directions) material law to describe the elastic properties of the cell wall. This allowed us to study the effect of increased stiffness in circumferential directions due to oriented cellulose deposition (Sieberer *et al.*, 2009). Since neither varying the speed of indentation (reduced by half) nor repeating each experiment three times in a row significantly affected force-indentation curves, we neglected any viscoelastic or plastic material properties. The constitutive equations can be written with respect to a local co-ordinate system that we defined to point in-plane in a circumferential direction (direction *r*), in-plane longitudinal (direction *l*), and in the thickness direction (direction *t*, normal to the cell wall) at each point of the shell (Fig 1B). The constitutive equations could then be written as a linear relation between true strains *ε*_*ij*_ and Cauchy stresses *σ*_*ij*_. The material parameters included three Young’s moduli, *E*_*r*_, *E*_*l*_, and *E*_*t*_, that controlled elasticity along each material direction, six Poisson’s ratios, *υ*_*rl*_, *υ*_*lr*_, *υ*_*rt*_, *υ*_*tr*_, *υ*_*lt*_, and *υ*_*tl*_, that controlled the Poisson effect between directions, and three shear moduli, *G*_*rl*_, *G*_*rt*_, and *G*_*lt*_, that controlled shear elasticity. Since the compliance matrix must be symmetric, three of the Poisson’s ratios depended on the Young’s moduli and the other Poisson’s ratios were constrained by the symmetry condition *υ*_*ij*_/*E*_*i*_=*υ*_*ji*_/*E*_*j*_, leaving nine independent material parameters. Given our current knowledge on cell wall material, there are still too many degrees of freedom. We thus assumed that the material was fully incompressible, thereby further constraining the relationship between the Young’s moduli and Poisson’s ratios (Itskov and Aksel, 2002), and chose to keep *E*_*l*_, *E*_*r*_, and *υ*_*rl*_ as independent constitutive parameters for the tension-compression part of the compliance matrix. This may seem like a strong assumption; therefore, the sensitivity of the results to this assumption was tested specifically. A last simplification was to assume that all the shear moduli *G* were the same. This led to a material model with four degrees of freedom, and we use *E*_*r*_, *E*_*l*,_ *υ*_*rl*_, and *G* to characterize the cell wall material. The interior of the cell was treated as a fluid-filled cavity that exerts a hydrostatic pressure *P* on the cell wall. Either the pressure or the volume of the cavity could be assigned a fixed value but never both at the same time.

The simulation was divided into two quasi-static steps (Fig. 1B). In the first step, the unloaded cell was pressurized by imposing turgor pressure within the cavity. This caused the cell to increase its volume and build up mechanical stress in the cell wall. In the second step, a hemispherical probe vertically indented the cell, which was supported by a plane underneath. To obtain smooth force versus indentation curves, we divided this step into 34 increments of the probe displacement. The interaction between the probe and the cell, and between the cell and the supporting plane, was modelled by frictionless contact. We expected this choice to have minor influence on simulated reaction forces because the boundary conditions prevented substantial sliding. For the indentation step, we compared two limiting assumptions on the water movements between the cell and its surroundings (Fig. 1C–E). ‘Constant pressure’ described the situation where any potential increase in hydrostatic pressure due to the indentation was immediately compensated by water outflow. We implemented this condition by holding the pressure within the cavity to a fixed, initial value. ‘Constant volume’ described the opposite case where water could not pass the wall within the time scale of a CFM experiment, and in this case turgor pressure increased during indentation.

All simulations were performed in Abaqus Standard 6.12 (DS Simulia). Because the cell, indenter, and boundary conditions were all symmetrical about both the central vertical and horizontal planes, only a quarter of the cell was modelled. The cell wall was discretized into approximately 1500 fully integrated, linear shell elements, using a large strain and large deformation formulation. We graded the mesh density to obtain higher resolution around the area of contact and performed a mesh sensitivity analysis to guarantee a small discretization error (Supplementary Fig. S4 at *JXB* online). The nodes of the shell elements were also used to define the fluid cavity. To verify the implicit computation of material parameters, we compared the simulated deformation during inflation against the analytic solution for the stress versus strain distribution in an anisotropic, cylindrical pressure vessel (see Eqs 2 and 3 below). Note that shell elements cannot model the compression of the contact patch or the resulting Poisson effect according to *υ*_*tl*_ and *υ*_*tr*_, nor any out-of-plane shear. This choice was thus consistent with the previous assumptions of the negligibility of the out-of-plane coefficients of the compliance matrix describing the constitutive elastic equations of the wall material.

### Dimensional analysis

During each finite-element simulation, equilibrium stresses and strains were computed for a sequence of increasing indentation depths (Fig. 1C). The corresponding reaction force that acted on the probe was part of each solution and can be thought of as a function of the model parameters:

$$f=\Phi \left(P\text{, }E_{r}\text{, }E_{l}\text{, }\nu_{rl}\text{, }G\text{, }r\text{, }l\text{, }t\text{, }s\text{, }x\right)$$ (1)

where *P* is the turgor pressure, *r* is the radius of the pressurized cell, *l* is the length of the unpressurized cell, *t* is the thickness of the cell wall, *s* is the radius of the probe, and *x* is the indentation depth (see Fig. 3A). The parameters in Eq. 1 can be classified into purely mechanical and purely geometrical parameters. This is useful for the theoretical study of the problem; however, in practice none of the mechanical properties of *P*, *E*_*r*_, *E*_*l*_, *G*, and *υ*_*rl*_ can be measured easily. We therefore also used the following alternative parameterization defining two dimensionless numbers, *ε*_*r*_ and *ε*_l_:

$$\varepsilon_{r}\text{:}=\frac{Pr}{t}\left(\frac{\text{1}}{E_{r}}-\frac{\nu_{rl}}{\text{2}E_{l}}\right)$$ (2)

and,

$$\varepsilon_{l}\text{:}=\frac{Pr}{t}\left(\frac{-\nu_{rl}}{E_{r}}+\frac{\text{1}}{\text{2}E_{l}}\right)\text{.}$$ (3)

This definition is motivated by the stress distribution in an orthotropic, thin-walled, cylindrical pressure vessel. In this case, *ε*_*r*_ and *ε*_*l*_ correspond to the equilibrium strain upon pressurization in the circumferential and longitudinal directions. These pre-strains are direct observable quantities that can be measured with a microscope by releasing turgor pressure with osmotic treatments and observing the subsequent shrinkage of the cell in both directions. We also re-parametrized shear elasticity by:

$$\beta \text{:}=\frac{\text{3G}}{E_{l}}\text{.}$$ (4)

Note that for *β*=1, the shear modulus *G* is the same as for an isotropic material with a Young’s modulus *E*_*l*_ and a Poisson’s ratio *υ*=0.5. The value of *β* is unknown for the primary plant cell wall; however, studies on other orthotropic cell wall material (i.e. from dry wood) would place *β* in the range 1<*β*<3 (Neagu and Gamstedt, 2007; Jäger *et al.*, 2011). In simulations, we thus chose *β*=2.

Using this alternative parameterization the dimensionless reaction force can be written as:

$$\frac{f}{Pr^{\text{2}}}=\hat{\Phi }\left(\frac{l}{r}\text{, }\frac{t}{r}\text{, }\varepsilon_{r}\text{, }\varepsilon_{l}\text{, }\upsilon_{rl}\text{, }\beta \text{, }\frac{s}{r}\text{, }\frac{x}{r}\right)\text{.}$$ (5)

According to a dimensional analysis, and in particular the Buckingham π theorem (Sonin, 2001), Eq. 5 is equivalent to Eq. 1.

### CFM indentation of tobacco BY-2 cells

**Fig. 2.**
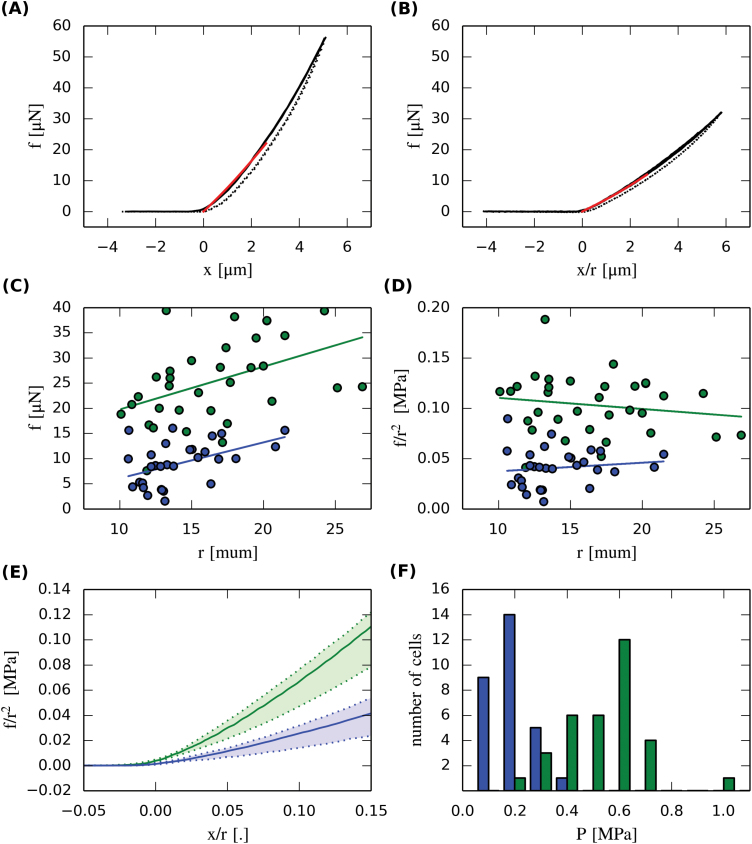
CFM measurements and pressure estimates. (A, B) CFM data for a BY-2 cell in water (A) and for another cell in 0.2M mannitol solution (B). Solid lines represent the movement into the sample and dashed lines denote the retraction phase. Red lines denote the model fit to the experimental data. (C) The force at *x*=2.3 μm was correlated with radius under both osmotic conditions [green=0M, blue=0.2M in (C)–(E)]. (D) This correlation disappeared when we rescaled force and indentation and reported values at *x*/*r*=0.15. (E) Summary of all CFM data. Solid lines denote the median of each group and the shaded area covers the 25–75% percentile. (F) Histogram of turgor pressure obtained by combining CFM, osmotic treatments, and inverse finite-element modelling.

Previous experiments on onion epidermal cells showed that the apparent stiffness from CFM measurements can strongly depend on turgor pressure (Routier-Kierzkowska *et al.*, 2012). In order to determine how sensitive CFM measurements on tobacco BY-2 cells are to turgor pressure, we performed indentation experiments on asynchronous cells that were submerged in either 0.2M mannitol or pure water for at least 30min. We measured the radius of each cell and set the maximum indentation depth to at least *x*/*r*=0.16. We then recorded the force versus indentation relationship by using a CFM probe of approximately 1 µm tip diameter, with a loading rate of about 1 µm s^–1^. As we expected from previous experiments on onion epidermis, BY-2 cells required significantly less force to indent by the same amount in 0.2M mannitol than in water (Fig. 2A, B, E). We also observed a positive correlation between cell radius and the force measured at the same absolute indentation of 2.3 μm (Fig. 2C). This correlation disappeared when we rescaled force by the radius squared and indentation by radius (Fig. 2D). This showed that the cell radius is an important parameter that needs to be measured when doing micro-indentation.

### Pre-strain and osmotic potential of tobacco BY-2 cells

When a cell is turgid, the cell wall is in a state of tensional strain that is characterized by the magnitude of *ε*_*r*_ and *ε*_*l*_. Previous studies on tomato protoplasts have shown that pre-strain can have a significant effect on micro-indentation data (Wang *et al.*, 2004). For this reason, we measured the strain for the two osmotic conditions that were used during CFM indentation. One group of cells was kept in 0.2M mannitol solution (normal osmolarity of medium) and another in water before plasmolysing both of them in 0.55M mannitol solution. This treatment caused an average longitudinal shrinkage equivalent to *ε*_*l*_=0.048 (std=0.051, *n*=39) when starting from 0.2M mannitol and *ε*_*l*_=0.082 (std=0.04, *n*=29) when starting from water. Shrinkage *ε*_*r*_ in the circumferential direction was considerably less, indicating that the cell was stiffer in this direction. Measurement sensitivity was reduced in the radial direction since the absolute amount of shrinkage was smaller than in longitudinal direction. Since the cell walls appeared approximately 1.5 μm thick due to shadowing effects, shrinkage under 5% in the radial direction was difficult to quantify.

In order to gain a rough idea of turgor pressure in BY-2 cells, we performed an incipient plasmolysis test. Incipient plasmolysis is defined as the osmotic condition where 50% of the cells are plasmolysed. At this point, the osmotic potential inside the cell matches the osmotic potential of the medium on average. We found incipient plasmolysis to happen at between 0.4 and 0.425M mannitol (*n*=55, Supplementary Fig. S3 at *JXB* online), which corresponded to an osmotic potential of about –1.0MPa (calculated by van’t Hoff’s equation) at room temperature. If the osmotic potential is assumed to be constant within a cell, it can be used to predict turgor pressure. This led to an estimated turgor pressure of about 1MPa for cells in water and 0.5MPa for cells in 0.2M mannitol. We expect these values to overestimate the true turgor pressure because plant culture cells are likely to be able to adapt their internal osmotic potential to the environment.

### Parameter sensitivity analysis

The previous experiments showed that CFM is sensitive to turgor pressure and cell radius. We then asked which other variables also significantly affect micro-indentation measurements? To answer this question, we performed a parameter sensitivity analysis of Eq. 1. We chose a set of representative parameters and then varied each of those parameters one at a time, under the assumptions described earlier (e.g. constant cell volume). The results are presented as relative sensitivity of the dimensional contact force Φ (Eq. 1) to each argument *x*_*i*_ as:

$$\psi_{x_{i}}=\frac{\partial \Phi }{\partial x_{i}}\frac{x_{i}}{\Phi }{|}_{x_{\text{ref}}}\text{,}$$ (6)

where *x*_ref_ denotes a vector of reference parameters. The relative sensitivity has the following intuitive meaning. For pressure, Ψ_*P*_ describes how much the relative reaction force increases for a relative increase in turgor pressure *P*. Thus, parameters that correspond to a high relative sensitivity have a strong effect on the reaction force. Parameters with high sensitivity need to be measured precisely when used as an input parameter of the model; however, they can be fitted more robustly to experimental data when treated as free parameters.

It can be shown that, given a set of fixed dimensionless parameters (*l*/*r*, *t*/*r*, *x*/*r*, *s*/*r*, *β*, *υ*_*rl*_, *ε*_*r*_, and *ε*_*l*_) of Eq. 5, there is a unique relative sensitivity for each of the physical parameters (*P*, *l*, *r*, *t*, *x*, *s*, *G*, *υ*_*rl*_, *E*_*r*_, and *E*_*l*_) of Eq. 1 about a particular reference state. We studied the relative sensitivity of physical parameters (Eq. 1) at eight different reference states, which are characterized by *l*/*r*=150/15, *t*/*r*=0.25/15, *β*=2, and *υ*_*rl*=_0.4, the initial strains being either low (*ε*_*r*=_0.005, *ε*_*l*=_0.01) or high (*ε*_*r*=_0.05, *ε*_*l*=_0.1), and the probe being either small (*s*/*r*=0.5/15) or large (*s*/*r*=15/15). We computed sensitivities for both shallow indentation (*x*/*r*=0.7/15) and larger depth (*x*/*r*=2.4/15), the last case being comparable to maximal indentation used in our experiment. The results are shown in Table 1. Note that the values for *l* and *r* are based on optical microscopy, the probe size *s* is provided by the manufacturer, the cell wall thickness is based on measurements for other thin-walled cells (Rezvani Moghaddam and Wilman, 1998), and the remaining parameters should represent a material that is about four times stiffer in the circumferential than in the longitudinal direction, although the exact amount depends on the Poisson effect.

**Table 1.** Relative sensitivities Ψ of the contact force to the parameters of Eq. 1 Sensitivities are shown for stiff cells walls (or low turgor pressure) and soft cells walls (or high turgor pressure) and two indenter sizes as well as two indentation depths. The small indenter corresponds to our experimental set-up, whereas the large indenter is similar in size to that found in ball tonometry setups. Large indentation depth was used in our experiments (light grey columns). The dark grey column approximates the reference configuration used for the interpretation of the experiments on BY2 cells.

	Stiff cell wall or low turgor (*ε*_*r*_=0.005, *ε*_*l*_=0.01)				Soft cell wall or high turgor (*ε*_*r*_=0.05, *ε*_*l*_=0.1)			
	Small indenter (*s*/*r*=0.033)		Large indenter (*s*/*r*=1.0)		Small indenter (*s*/*r*=0.033)		Large indenter (*s*/*r*=1.0)	
	*x*/*r*=0.7/15	*x*/*r*=2.4/15	*x*/*r*=0.7/15	*x*/*r*=2.4/15	*x*/*r*=0.7/15	*x*/*r*=2.4/15	*x*/*r*=0.7/15	*x*/*r*=2.4/15
Indentation depth (*x*)	0.96	1.20	1.06	1.18	1.04	1.11	1.15	1.18
Pressure (*P*)	0.67	0.69	0.77	0.79	0.79	0.75	0.81	0.84
Cell radius (*r*)	0.31	0.36	0.25	0.28	0.57	0.50	0.42	0.36
Wall thickness (*t*)	0.68	0.43	0.43	0.29	0.33	0.29	0.22	0.17
Young’s modulus longitudinal direction (*E*_*l*_)	0.14	0.17	0.08	0.12	0.08	0.12	0.05	0.06
Probe radius (*s*)	0.01	0.02	0.21	0.27	0.06	0.11	0.20	0.26
Young’s modulus transverse direction (*E*_*r*_)	0.10	0.06	0.09	0.05	0.06	0.07	0.06	0.05
Shear modulus (*G*)	0.09	0.08	0.06	0.04	0.07	0.06	0.06	0.05
Poisson ratio (*υ*_*rl*_)	0.03	0.02	0.02	0.02	0.01	0.02	0.005	0.01
Cell length (*l*)	0.04	–0.01	0.05	–0.03	–0.01	0.01	–0.01	0.02

### Sensitivity to mechanical parameters

As shown in Table 1, the mechanical parameter with the highest influence on reaction force was turgor pressure (0.67<Ψ_*P*_<0.83) for all conditions. Even for the small pre-strain case (*ε*_*r*_=0.005, *ε*_*l*_=0.01), which can illustrate a cell with relatively low turgor, the internal pressure was several times more important than any single mechanical property of the cell wall. Still, the sensitivity to *E*_*r*_, *E*_*l*_, and *G* was not negligible, showing that we did not indent deep enough to be in the purely pressure-dominated limit case proposed by Vella *et al.* (2012*b*). The relative sensitivity to *E*_*l*_ was substantially higher than for *E*_*r*_ in all except the large pre-strain, large probe case. Interestingly, the relative sensitivity to the circumferential Young’s moduli *E*_*r*_ depended little on probe size and pre-strain, whereas the relative sensitivity for *E*_*l*_ did. The Poisson’s ratio *υ*_*rl*_ was found to have a negligible effect, under our assumption of full incompressibility. For the range of indentations we considered, the sensitivities to pressure and Young’s moduli did not differ significantly between shallow and deeper indentation cases, reflecting the fact that we were in a roughly linear regime of indentation.

Beside the parameters in Eq. 1, we also studied the sensitivity of the contact force to the hydraulic conductivity of the cell wall. We compared the relative change in force at the four reference states when using the constant pressure instead of the constant volume assumption. The constant pressure simulations showed a decreased reaction force by a maximum of 5.3% in the small pre-strain, large probe case. For a small probe, this difference was significantly smaller (<3%), showing that water transport induced by squeezing the cell could be neglected in our experiments (Table 2), where the average indentation time was approximately 10 s. Table 2 also shows that the reaction force had little sensitivity to the cell wall compressibility assumption described previously.

**Table 2.** Effect of assumptions of water movements and cell wall compressibility on the reaction force (%) Shading is as in Table 1.

	Stiff cell wall or low turgor (*ε*_*r*_=0.005, *ε*_*l*_=0.01)				Soft cell wall or high turgor (*ε*_*r*_=0.05, *ε*_*l*_=0.1)			
	Small indenter (*s*/*r*=0.033)		Large indenter (*s*/*r*=1.0)		Small indenter (*s*/*r*=0.033)		Large indenter (*s*/*r*=1.0)	
	*x*/*r*=0.7/15	*x*/*r*=2.4/15	*x*/*r*=0.7/15	*x*/*r*=2.4/15	*x*/*r*=0.7/15	*x*/*r*=2.4/15	*x*/*r*=0.7/15	*x*/*r*=2.4/15
Constant total cell volume vs constant pressure	0.4%	3%	0.6%	5%	0.4%	1%	0.6%	1%
Fully incompressible vs fully compressible cell wall	1.9%	0.9%	1.3%	1%	0.2%	2.4%	0.5%	0.5%

### Sensitivity to geometrical parameters

From the geometrical parameters, the indentation depth was found to be the most important parameter (1.1<Ψ_*x*_<1.2; Table 1). Thus, a precise control of the robot positioner and detection of the contact point is crucial when interpreting absolute force values from micro-indentation experiments. Another important parameter was the radius of the deformed cell (0.25<Ψ_*r*_<0.57). This reflects the fact that the pressure-induced cell wall stresses, which are transmitted to the probe, are proportional to the radius. The effect was smaller for large probe sizes, most likely because less pre-stress is transferred to the probe due to a flat contact geometry. In case of deeper indentations, we also found a moderate influence of cell wall thickness (0.17<Ψ_*t*_<0.43), which is linked to the overall stiffness of the shell. In particular, for the same material parameters *E*_*r*_, *E*_*l*_, *G*, and *υ*_*rl*_, and for the same pressure, an increased thickness caused a decrease in *ε*_*r*_ and *ε*_*l*_. In this sense, the relative sensitivity to thickness can be viewed as the relative sensitivity to the sum of all elastic properties. Beside tensional stiffness, the thickness also affected the bending stiffness, leading to a higher Ψ_*t*_ for a small probe and low pre-strain where bending was expected to have more influence. The exact radius of the probe did not matter if it was small. In this case, it could be replaced by a point load in the model with little impact on the accuracy of the model. Finally, we found only a small sensitivity to the length of the cell (|Ψ_*l*_|<0.05), showing that at *l*/*r*=10, the cell already behaved like an infinite cylinder.

### Alternate parameterization sensitivity

**Fig. 3.**
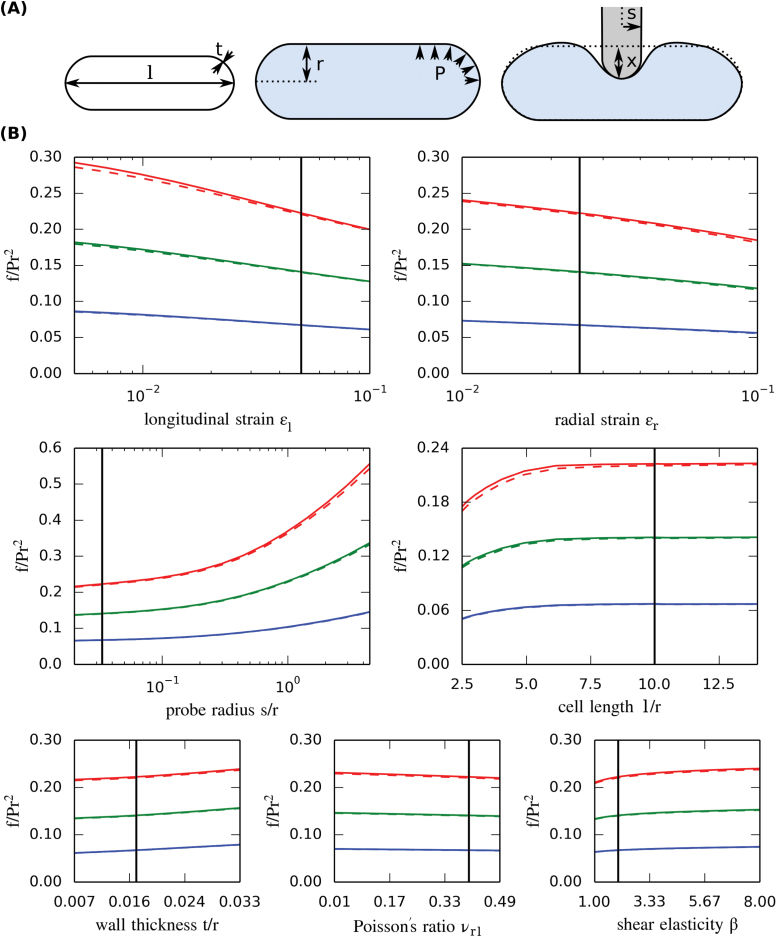
Finite-element sensitivity analysis. (A) Illustration of parameters in the model. (B) Reaction force obtained when varying one input parameter at a time around a common reference state (*l*/*r*=150/15, *t*/*r*=0.25/15, *x*/*r*=2.4/15, *s*/*r*=0.5/15, *ε*_*r*_=0.025, *ε*_*l*_=0.05, *β*=2, *υ*_*rl*=_0.4). Colours refer to indentation depths of *x*/*r*=0.05 (blue), *x*/*r*=0.1 (green), and *x*/*r* =0.15 (red). Simulations are performed assuming either constant volume (solid lines) or constant pressure (dashed lines) during the indentation step. The black line indicates the reference value.

The sensitivity analysis presented so far in Eq. 1 concerned parameters such as pressure, Young’s modulus, and Poisson’s ratio that are readily controllable in simulations but cannot be directly measured experimentally. On the other hand, parameters such as *ε*_*r*_ and *ε*_*l*_, the pre-strains due to turgor pressure (Eqs 2 and 3), are direct observable quantities that can be measured as the cell deforms upon turgor pressure release due to osmotic treatment. We therefore used Eq. 5, the dimensionless equivalent of Eq. 1, to study the sensitivity of the dimensionless force (f/P*r ^2^) to parameters such as *ε*_*r*_ and *ε*_*l*_, as well as purely geometric parameters such as the ratios *l*/*r* or *t*/*r*. This sensitivity analysis (Table 3, Fig. 3) enabled us to identify the parameters that are relevant for the interpretation of our experiments and had the advantage of being scalable to any size of cell. From the parameters of Eq. 5 that depend on mechanical properties of the cell wall, we found the strain in the longitudinal direction *ε*_*l*_ to have a moderate influence on the reaction force. In contrast, the strain in the radial direction *ε*_*r*_ and the dimensionless shear modulus *β* had little influence on the reaction force. Varying *ε*_*r*_ by an order of magnitude only changed the reaction force by approximately 25%. The Poisson’s ratio (in-plane between the radial and longitudinal directions) *υ*_*rl*_ was found to have a negligible influence. From the geometrical parameters, we found the exact length of the cell *l*/*r* to be negligible for *l*/*r*>7. The same held for the probe radius *s*/*r* as long as it was small.

**Table 3.** Relative sensitivities Ψ of the contact force to the parameters of Eq. 5. Eight reference configurations are shown, as in Table 1. Shading is as in Table 1. Note that the relative sensitivity for wall thickness (*) is very different from the value given in Table 1

	Stiff cell wall or low turgor (*ε*_*r*_=0.005, *ε*_*l*_=0.01)				Soft cell wall or high turgor (*ε*_*r*_=0.05, *ε*_*l*_=0.1)			
	Small indenter (*s*/*r*=0.033)		Large indenter (*s*/*r*=1.0)		Small indenter (*s*/*r*=0.033)		Large indenter (*s*/*r*=1.0)	
	*x*/*r*=0.7/15	*x*/*r*=2.4/15	*x*/*r*=0.7/15	*x*/*r*=2.4/15	*x*/*r*=0.7/15	*x*/*r*=2.4/15	*x*/*r*=0.7/15	*x*/*r*=2.4/15
Indentation depth (*x*/*r*)	0.95	1.20	1.06	1.19	1.04	1.10	1.16	1.18
Longitudinal pre-strain (*ε*_*l*_)	–0.21	–0.22	–0.13	–0.14	–0.12	–0.14	–0.10	–0.09
Radial pre-strain (*ε*_*r*_)	–0.14	–0.11	–0.12	–0.08	–0.10	–0.10	–0.10	–0.08
Probe radius (*s*/*r*)	0.01	0.02	0.21	0.27	0.07	0.10	0.20	0.26
Shear modulus (*β*)	0.09	0.08	0.06	0.04	0.07	0.06	0.06	0.05
Wall thickness (*t*/*r*)*	0.35	0.12	0.2	0.09	0.12	0.04	0.03	0.01
Poisson ratio (*υ*_*rl*_)	–0.05	–0.06	–0.04	–0.03	–0.04	–0.04	–0.04	–0.03
Cell length (*l*/*r*)	0.04	–0.01	0.05	–0.03	–0.01	0.01	0.01	0.02

Strikingly, the effect of cell wall relative thickness (*t*/*r*) was negligible (Table 3). This is in contrast to the analysis done with Eq. 1 (Table 1). In the dimensionless parameterization of Eq. 5, the elastic parameters are contained in the two (measurable) strains, and thus an increase in wall thickness reduces the Young’s moduli so that the same strains are maintained (see Eqs.2 and 3). This means that, although the total stiffness of the wall (i.e. the product of Young’s modulus *E* by thickness *t*) is important, the thickness *per se* is not (Wang *et al.*, 2004). The reason is that *t*/*r* in this parameterization has no effect on the tensional but only on the bending stiffness of the shell in Eq. 5, and since the wall is thin, the bending stiffness is negligible.

### Reverse-engineering turgor pressure in BY-2 cells

Eq. 5 can be used to calculate turgor pressure by solving an inverse problem. The idea is to find the model parameter *P* that best fits the CFM and osmotic shrinkage data. We ran one finite-element simulation for each osmotic condition by using the corresponding average longitudinal pre-strain (i.e. *ε*_*l*_=0.048 and *ε*_*l*_=0.082). The other parameters were set to *l*/*r*=7, *ε*_*r*_=*ε*_*l*_ /2, *β*=2, *υ*_*rl*=_0.4, *t*/*r*=0.25/15.0, *x*/*r*=0.15, and *s*/*r*=0.5/15. Since Φ is not very sensitive to these parameters, we expected this choice to have little effect on the estimates of *P*. The finite-element simulations resulted in two values of Φ, one for each osmotic condition. From these values, we calculated turgor pressure for each cell by dividing the rescaled contact force *f*/*r* ^2^ at *x*/*r*=0.15 by Φ (Fig. 2F). We found turgor pressure to be 0.18±0.08MPa (mean±standard deviation) for cells in 0.2M solution and 0.54±0.15MPa for cells in water (Fig. 2F). These values are consistent with the range of turgor pressures that has been reported for larger plant cells using a pressure probe (Tomos and Leigh, 1999) but ~50 % smaller than estimates that were based on the incipient plasmolysis experiment. This indicates that the concentration of osmolytes in the cell decreases with decreasing molarity of the medium, suggesting osmoregulation by the cells.

We also made a rough comparison of our results to the asymptotic formulas given by Vella *et al.* (2012*b*). These formulas cover two linear regimes when indenting a pressurized, isotropic, ellipsoidal shell fixed at the equator with a point load. The first formula applies to very small indentations, while the second applies to very large indentations. Assuming an isotropic and fully compressible material (*E*_*r*_=*E*_*l*_=300MPa, *t*=0.25 μm, *r*=15 μm, *P*=0.5MPa, *υ*_*rl*_=*υ*_*rt*_=*υ*_*lt*_=0) and fixing the equator after pressurization, we found that the force versus indentation curves predicted by our model were better approximated by the formula for small indentations than by the formula for large indentations, although quantitatively our situation is somewhere in between.

### Estimating elasticity in BY-2 cells

Once the pressure was known, Eqs 2 and 3 could be used to recover the Young’s moduli *E*_*r*_ and *E*_*l*_. In contrast to the pressure, *E*_*r*_ and *E*_*l*_ strongly depended on *ε*_*r*_, *t*, and *υ*_*rl*_. Taking the average pressure *P* and pre-strains *ε*_*l*_ in water, and by setting *t*/*r*=0.25/15.0, *ε*_*r*_=*ε*_*l*_/2, and *υ*_*rl*=_0.4 in the model, we obtained an estimated Young’s moduli for the whole population of tobacco cells we measured of *E*_*r*_=591±166MPa and *E*_*l*_=153±43MPa. Using the average pressure and pre-strain at 0.2M resulted in lower values for the population’s Young’s moduli in both directions (*E*_*r*_=365±164MPa, *E*_*l*_=91±41MPa). This means that the elastic modulus increases with increasing strain, indicating a strain-stiffening behaviour of the cell wall (Deng *et al.*, 2011; Kierzkowski *et al.*, 2012). Note that, in contrast to the estimates of turgor pressure, these estimates do depend on the choice of thickness *t*, and the Poisson’s ratio *υ*_*rl*_. Additionally, as we could not resolve the measurement of the radial pre-strain *ε*_*r*_ in our osmotic experiments, our estimates of the anisotropic ratio *E*_*r*_/*E*_*l*_ were dependent on the assumption concerning the pre-strain ratio (i.e. *ε*_*r*_=*ε*_*l*_/2).

### Practical use of sensitivity analysis in CFM experiments

Sensitivity analysis can be used to design experiments by identifying which parameters have the most influence on the measured force, and in particular cases to provide a quick interpretation of experimental results. For example, if the pre-strain parameters (*ε*_*r*_ and *ε*_*l*_) measured by osmotic treatment of two cells of the same radius are the same, it can be deduced from Eq. 5 that a change in force is directly proportional to a difference in turgor pressure, given the same indentation depth. In the case when the pre-strain is slightly different, the outcome would be very similar, since the measured force is not very sensitive to *ε*_*r*_ and *ε*_*l*_ (Table 3, Fig. 3B). If the cells have different radii, the interpretation would not be so straightforward. Eq. 5 shows that, for constant Φ (i.e. all parameters being the same, except for pressure *P* and absolute cell radius *r*), the measured force is proportional to both the pressure and cell radius squared, assuming a fixed relative indentation depth. In contrast, if the cells have a different size with all other dimensionless parameters being constant (‘scaled’ cells), for a fixed absolute indentation depth the dimensionless force of Eq. 5 is almost proportional to the relative indentation (*x*/*r*) for the range of depths considered (Table 3, Fig. 2E):

$$\frac{f}{Pr^{\text{2}}}\propto {\left(\frac{x}{r}\right)}^{\text{1.10}}$$

At a fixed absolute indentation depth *x*, the pressure is therefore roughly proportional to the measured force, divided by cell radius:

$$P\propto \frac{f}{r^{\text{0.90}}}$$

Caution should be taken when applying this method in that only one important parameter should be varied at a time. In the case when several sensitive parameters vary, such as relative indentation depth *and* pre-strain, Eq. 5 has to be solved for a particular set of parameters in order to reverse-engineer the pressure. It should be also stressed that the sensitivity values given in Tables 1 and 3 are valid only for the particular geometry of BY2-cells. Nonetheless, once such a sensitivity analysis is provided for a given set of experiments, it is a valid technique for ‘rule of thumb’ evaluations.

## Discussion

The mechanical aspects of morphogenesis are increasingly being explored through the use of new automated indentation methods. Although all indentation methods share the same principle, they operate in different parameter ranges, and thus differ in sensitivity towards the various physical properties of the cell. In the case of larger indentations (comparable to cell wall thickness and larger), the reaction force is composed of three parts. The first is due to the bending stiffness of the cell wall, which is proportional to the Young’s modulus and the third power of thickness for an isotropic material (Vella *et al.*, 2012*b*). If pressure is zero, bending stiffness dominates the reaction force. This effect rapidly decreases as the cell becomes turgid and pressure dominates the measurement. The second component of the reaction force comes from tension within the cell wall. As the cell is indented, in-plane tension aligns more with the axis of indentation causing the load to transfer onto the probe. Some of the tension is present from the beginning due to turgor pressure and some of it is induced by stretching the material during indentation. The low sensitivity to cell wall elasticity and the high sensitivity to pressure suggest that in our case pressure-induced tension plays an important role, whereas the additional indentation induced tension does not. The third contribution to reaction force is the pressure which is ‘directly’ transmitted through the wall by compressing the contact patch. This contribution increases with increasing contact area and therefore depends on probe size and shape. These parameters have little influence if the probe size is small. If the probe size is very large, however, this contribution can dominate the reaction force (Lintilhac *et al.*, 2000; Wang *et al.*, 2006). Note that for very small indentations, there is also a contribution from compressibility of the wall itself. This would be significant during very small indentation depths (nanoindentation), as can be performed by AFM (Milani *et al.*, 2011), although the larger displacement and force ranges of CFM are probably unable to detect this.

In general, all three components may have some contribution to a micro-indentation experiment. Thus in order to extract physical properties from force versus indentation data, a mechanical model that can simulate all of these effects is required. Such a model can be derived within the framework of continuum mechanics and be solved by the finite-element method. The more realistic these models are, the more parameters they include. This can cause problems if more than one parameter needs to be fitted to the experimental data or if some input parameters cannot be measured precisely. In this case, the parameter fitting might result in uncertain estimates or there may not be a unique solution.

Here, we have used the following approach to address these problems. First, a mechanical model of micro-indentation on a turgid BY2 cell was developed. The model features a realistic geometry and an anisotropic material definition of the cell wall. We then studied how much the simulated force-indentation relationship depends on individual parameters of the model. We showed that, for cells that are at least mildly turgid, the reaction force on the probe depends strongly on the indentation depth, the cell radius, and the pressure within the cell, a result that is supported by a similar study for spherical and ellipsoidal shells (Vella *et al.*, 2012*a*, *b*). The apparent stiffness increases for increasing cell radius in a turgid cell, whereas the opposite was shown for a non-pressurized cylindrical cell (Bolduc *et al.*, 2006). This difference can be explained by the transition from a bending stiffness to a pressure-dominated sample stiffness. In an unpressurized cell, an increasing radius decreases the overall stiffness if the thickness is assumed to be constant. In a pressurized cell, however, the stress in the cell wall increases for increasing radius, which positively affects the sample stiffness.

The parameters that control cell wall elasticity, wall thickness, length (for *l*/*r*>7), indenter size (if small), and hydraulic conductivity of the cell wall were found to have little to almost no effect in this study. This result held true for a variety of initial strains, indentation depths, and probe sizes. This is different from the results of Forouzesh *et al.* (2013), who found a significant sensitivity to cell wall elasticity when modelling indentations on a pressurized spherical disk but qualitatively similar to the results of Vella *et al.* (2012*b*), who modelled the indentation of a pressurized ellipsoidal/cylindrical shell. We expect the difference in geometry and boundary conditions to be the most likely explanation. Our results differ from those of Bolduc *et al.* (2006) where the probe size was shown to have a significant effect. It appears that probe size is much less important when performing micro-indentation on turgid cells.

Since indenter size is relatively unimportant, our results suggest that using a model with a point load is sufficient. However, differences of our model compared with that of Vella *et al.* (2012*b*) and Forouzesh *et al.* (2013) show that boundary conditions are very important. The most important material parameters are *E*_*r*_, *E*_*l*_, and *υ*_*rl*_, since the thickness direction is small and shear in the model is relatively low. Thus, a transverse isotropic or even membrane formulation is likely to be sufficient (Bozorg *et al.*, 2014). A fully isotropic material model, however, is not suitable when the stretch ratios observed in osmotic treatment show considerable anisotropy.

From the sensitivity analysis, we concluded that the turgor pressure of BY2 cells can be fitted with good accuracy. This is because reaction force, indentation depth, and cell radius can be measured precisely with the CFM set-up. From the remaining parameters, only the initial pre-strains *ε*_*r*_ and *ε*_*l*_ were found to have a significant effect. These can be measured by averaging the osmotic shrinkage when inducing plasmolysis. Overall, this procedure requires one more experiment (osmotic shrinkage) than the procedure described by Forouzesh *et al.* (2013), where turgor pressure and the wall elasticity of leaf epidermal cells were fitted to indentation data alone. Since cell wall elasticity did not have a major effect in our model, using indentation alone is expected to be unreliable for BY2 cells. However, osmotic shrinkage experiments are an excellent complement to micro-indentation experiments, since they are very sensitive to cell wall elasticity. Thus, the combination of the two approaches with inverse mechanical modelling can be used to provide reliable estimates for both pressure and elasticity.

With improved imaging techniques, a better estimate for the radial stretch ratio should be possible, yielding more accurate insights into the anisotropy of the cell wall. By using more sophisticated models, our approach could be extended to multicellular tissues. This would allow the study of gradients of turgor pressure and cell wall elasticity at a cellular level, a key requirement in the investigation of the relationship between genetics, mechanics, and morphogenesis in plants.

## Supplementary data

Supplementary data are available at *JXB* online.

Supplementary Fig. S1. Repeated indentations (*n*=100) on a glass slide reveal the sensor internal stiffness (a). Repeated indentations (*n*=100) on a SI-traceable stiffness standard (stiffness=15.17 N m^–1^) show that the data acquisition/processing work is repeatable and accurate (b).

Supplementary Fig. S2. CFM measurements at three different points within the same tobacco BY2 cell.

Supplementary Fig. S3. Percentages of cells which were plasmolysed after submerging them for 15min in a particular mannitol solution.

Supplementary Fig. S4. Simulated contact force at *x*=2.4/15, *L*/*r*=150=15, *t*/*r*=0.25/15, *s*/*r*=0.5/15, *ε*_l_=0.05, *β*= 2, *υ*_*rl*_=0.4 for a varying relative element size (higher values correspond to a coarser mesh).

Supplementary Data

## Abbreviations:

AFM: atomic force microscopy
CFM: cellular force microscopy.
