# Supplementary material for: Measuring the mechanical properties of plant cells by combining micro-indentation with osmotic treatments
Source: J Exp Bot. 2015 Apr 7;66(11):3229–41. doi: 10.1093/jxb/erv135 (PMC4449541; doi:10.1093/jxb/erv135)
Supplement: Supplementary Data [file supp_66_11_3229__index.html]

Measuring the mechanical properties of plant cells by combining micro-indentation with osmotic treatments — Measuring the mechanical properties of plant cells by combining micro-indentation with osmotic treatments — Supplementary Data 

# Measuring the mechanical properties of plant cells by combining micro-indentation with osmotic treatments

## Supplementary Data

Data files

**Files in this Data Supplement:**

- Supplementary Data - Supplementary Data
