# Supplementary material for: Measuring the mechanical properties of plant cells by combining micro-indentation with osmotic treatments
Source: J Exp Bot. 2015 Apr 7;66(11):3229–41. doi: 10.1093/jxb/erv135 (PMC4449541; doi:10.1093/jxb/erv135)
Supplement: Supplementary Data [file supp_erv135_jexbot133165_file001.pdf]

Measuring the mechanical properties of plant cells by combining micro-indentation with osmotic treatments.
-
Supplemental Information

Alain Weber, Siobhan Braybrook, Michal Huflejt, Gabriella Mosca,
Anne-Lise Routier-Kierzkowska, Richard S. Smith

Post-processing of CFM data

For the post-processing of the data we only considered force vs. z-position data when moving into the sample (not when retracting the probe). This raw data was processed in five steps. First we corrected for sensor internal cantilever deflection, by indenting on glass and extracting the stiffness with a linear fit (Fig. S1A). Since glass is a stiff material we assumed the deflection happens exclusively within the sensor. We found sensor-stiffness to be about 200 N/m. Subsequently we subtracted force divided by 200 N/m from all z-position data. This correction typically increased the estimated sample stiffness by about 10%. As a second correction we calculated the stiffness of the sample medium meniscus. First, we performed a linear regression on the first 20% of indentation where the probe is not yet in contact with the sample. Second, we removed this trend from the whole fine approach. This correction was found to affect sample stiffness only by a small amount, because the stiffness of the medium is small ($<$0.2 N/m) compared to the stiffness of the sample. In a third step of data processing, we aligned the three iterations by fitting the force vs. corrected z-position data with a quadratic polynomial. We extracted the z-position of the contact point by treating it as a degree of freedom. This resulted in a total number of three degrees of freedom which we fitted by non-linear optimization (optimize.leastsq in Scientific Python). In particular we minimized the sum of squared residuals over the range of data which starts at the contact point and ends at the deepest z-position. We then used the contact point to align the three curves (Fig. 3A-B). Finally, indentation was divided by the radius of the cell and contact force by the radius squared to correct for the correlation between reaction force and cell radius.

CFM system validation

In order to validate our data-acquisition and processing workflow we did an indentation experiment on a SI-traceable stiffness standard (FS-C, SiMETRICS GmbH). The stiffness of the standard (15.17 N/m) is comparable to a BY2 cell. We used the same force sensor and experimental settings as in all our BY2 experiments. The probe was positioned at two points close to a dedicated imprint before performing 100 and 50 indentations respectively. This resulted in an average sample stiffness of 14.86 N/m and 14.84 N/m respectively (Fig. S1b). The post-processing of the CFM data data was slightly modified from the one used on BY2 cells. Since we did not observe a nonlinear relation, we extracted the contact point by fitting force vs. z-position curves with a linear instead of a quadratic polynomial. As in all our experiments we corrected z-position values for the sensor internal deflection which was derived from repeated indentations on a glass slide (Fig. S1a).

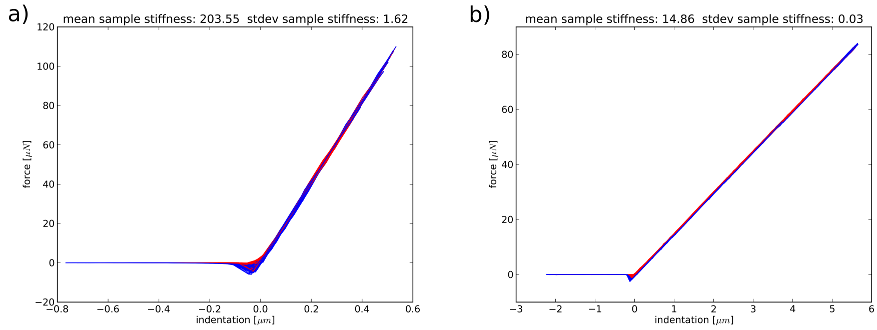

Fig. S1: Repeated indentations (n=100) on a glass slide reveal the sensor internal stiffness (a). Repeated indentations (n=100) on a SI-traceable stiffness standard (stiffness = 15.17 N/m) show that the data acquisition/processing workflow is repeatable and accurate (b). In both figures red lines denote the movement into the sample and blue lines the retraction phase.

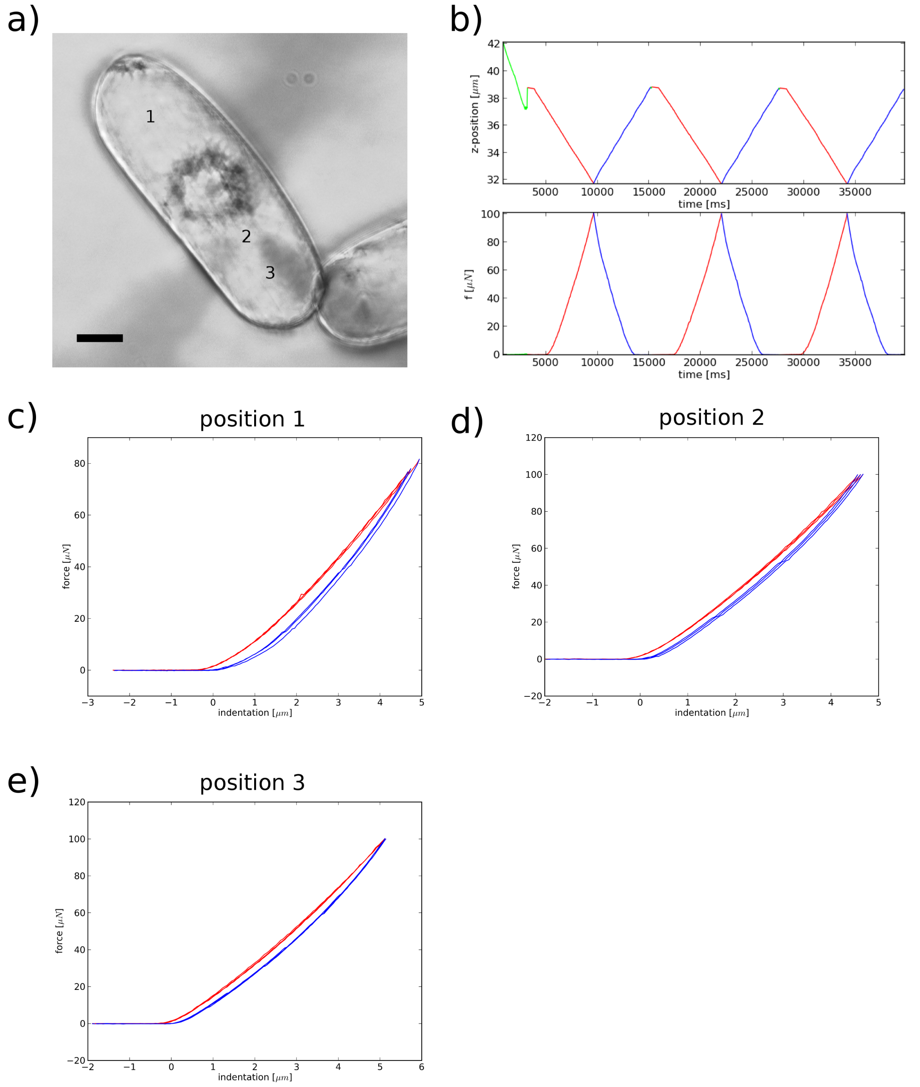

Fig. S2: CFM measurements at three different points within the same tobacco BY2 cell in water (a) (scale bar = $20\mu m$). As in all our experiments force indentation curves were constructed based on z-position vs. force measurements (b). The stiffness was lower near the ends of the cell (c,e) than close to the middle (d). In (b-e) red lines denote the movement into the sample and blue lines the retraction phase. While we do not observe hysteresis in the loading/unloading curves when indenting on glass (see Fig. S1), the slight difference observed in cell measurements indicates a viscoelastic effect. We chose to consider only the loading phase in our analysis. The green lines in (b) denote the coarse approach.

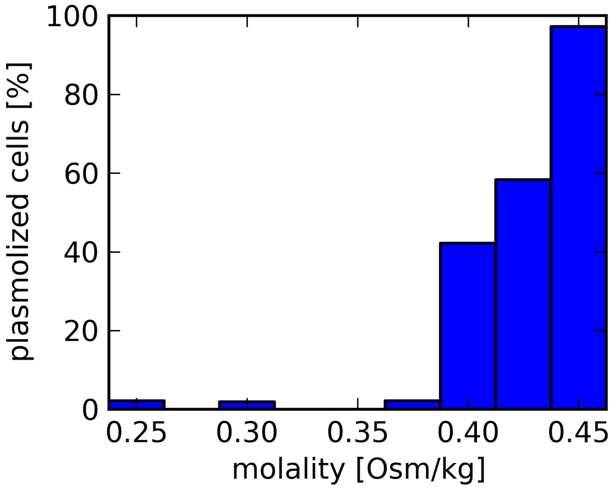

Fig. S3: Percentages of cells which were plasmolized after submerging them for 15 minutes in a particular mannitol solution. The point of incipient plasmolysis is defined as the molarity at which 50% of the cells are plasmolized (n=55).

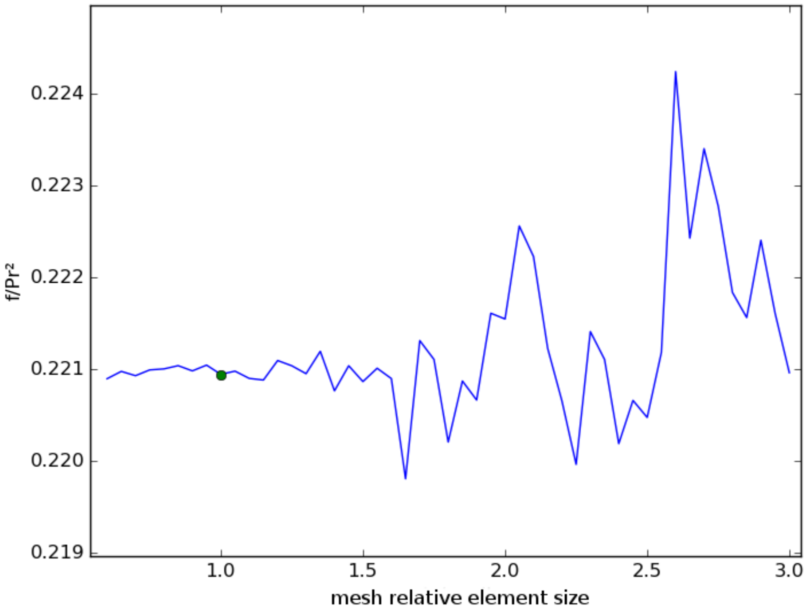

Fig. S4: Simulated contact force at $x = 2.4/15$, $L/r = 150/15$, $t/r = 0.25/15$, $s/r = 0.5/15$, $\epsilon_l = 0.05$, $\beta = 2$, $\nu_{rl} = 0.4$ for a varying relative element size (higher values correspond to a coarser mesh). The mesh density used in this study (green dot) offers a good trade-off between precision and computational time.
